# Metformin mediates induction of miR‐708 to inhibit self‐renewal and chemoresistance of breast cancer stem cells through targeting CD47

**DOI:** 10.1111/jcmm.14462

**Published:** 2019-07-05

**Authors:** Weige Tan, Hailin Tang, Xinhua Jiang, Feng Ye, Lili Huang, Dingbo Shi, Laisheng Li, Xiaojia Huang, Li Li, Xiaoming Xie, Xinhua Xie

**Affiliations:** ^1^ Department of Breast Surgery The First Affiliated Hospital of Guangzhou Medical University Guangzhou China; ^2^ Sun Yat‐sen University Cancer Center, State Key Laboratory of Oncology in South China, Collaborative Innovation Center for Cancer Medicine Guangzhou China; ^3^ Guangzhou School of Medicine Guangzhou Medical University Guangzhou China; ^4^ Department of Laboratory Medicine The First Affiliated Hospital of Sun Yat‐sen University Guangzhou China

**Keywords:** BCSCs, CD47, metformin, MiR‐708

## Abstract

Breast cancer stem cells (BCSCs) have been considered responsible for cancer progression, recurrence, metastasis and drug resistance. However, the mechanisms by which cells acquire self‐renewal and chemoresistance properties are remaining largely unclear. Herein, we evaluated the role of miR‐708 and metformin in BCSCs, and found that the expression of miR‐708 is significantly down‐regulated in BCSCs and tumour tissues, and correlates with chemotherapy response and prognosis. Moreover, miR‐708 markedly inhibits sphere formation, CD44^+^/CD24^−^ ratio, and tumour initiation and increases chemosensitivity of BCSCs. Mechanistically, miR‐708 directly binds to cluster of differentiation 47 (CD47), and regulates tumour‐associated macrophage‐mediated phagocytosis. On the other hand, CD47 is essential for self‐renewal, tumour initiation and chemoresistance of BCSCs, and correlates with the prognosis of breast cancer patients. In addition, the anti‐type II diabetes drug metformin are found to be involved in the miR‐708/CD47 signalling pathway. Therefore, our study demonstrated that miR‐708 plays an important tumour suppressor role in BCSCs self‐renewal and chemoresistance, and the miR‐708/CD47 regulatory axis may represent a novel therapeutic mechanism of metformin in BCSCs.

## 1 INTRODUCTION

Breast cancer is the most common cancer in women, and approximately 2.1 million women worldwide are newly diagnosed with breast cancer in 2018, with estimated 626 679 associated deaths.^1^ Recent studies have demonstrated that cancer stem cells (CSCs) are responsible for cancer chemoresistance and metastasis, and microRNAs (miRNAs) play important roles in the obtaining of CSCs properties such as self‐renewal, tumour initiation, and so on.^2^, ^3^ In breast cancer, small population of cells termed as CSCs or tumour‐initiating cells are highly enriched in CD44^+^/CD24^−^ population and are thought to trigger drug resistance and cancer metastasis.^4^, ^5^ Previous studies showed that miRNA‐708 can suppress cancer progressions in numerous types of cancers.^6^, ^7^, ^8^, ^9^, ^10^, ^11^ In breast cancer, miR‐708 overexpression inhibited cancer metastasis by targeting neuronatin.^6^ Despite these findings showing miR‐708 as a potential tumour suppressor, its function in breast cancer stem cells (BCSCs) has not been reported, and how miR‐708 is regulated and the detailed mechanism is still unknown. In addition, it is likely that there are additional targets of miR‐708 involved in the regulation of BCSCs chemoresistance and cancer metastasis.

Over the past decade, accumulating evidence shows that the blockade of immune checkpoint has attracted wide attention and made great progress in anti‐cancer treatment.^12^ Macrophage phagocytosis checkpoint has been demonstrated to be an interesting and promising therapeutic target. Cluster of differentiation 47 (CD47) is a ubiquitously expressed cell‐surface glycoprotein of the immunoglobulin superfamily that plays a critical role in evasion of immunological eradication.^12^, ^13^, ^14^ The overexpression of CD47 in various cancers is clinically correlated with patients’ poor prognosis.^15^, ^16^ The main mechanism of CD47‐mediated immune escape is that it can interact with signal regulatory protein‐alpha (SIRPα) on the surface of macrophages to ultimately blocking phagocytosis.^17^, ^18^, ^19^ Moreover, CD47 expression is highly increased in CSCs that is mediated by hypoxia‐inducible factor 1 and agents targeting CD47 may be developed to eradicate CSCs.^20^ Furthermore, we search for the literatures and find CD47 is the predicted target gene of miR‐708, which can be predicted by current target gene prediction softwares.^9^

In recent studies, it was reported that metformin, a biguanide anti‐diabetic drug for the type II diabetes, can selectively reduce the number of BCSCs and suppress tumour development.^21^ Moreover, the expression of PD‐L1 in tumour cells can be effectively inhibited by metformin.^16^ These findings imply that metformin may exert its antitumour effects through regulating immune‐related genes and signalling pathways.

Here, we report that miR‐708 is significantly down‐regulated in BCSCs and inhibits self‐renewal and chemoresistance of BCSCs in vitro and in vivo. Interestingly, metformin reduces the stem cells through miR‐708 mediated repression of the gene CD47, which is involved in type II diabetes development. Moreover, miR‐708 and CD47 regulate the phagocytosis of BCSCs by macrophages. Furthermore, our clinical data suggest miR‐708 and CD47 expression in primary breast cancer tissues is associated with patients’ prognosis and response to chemotherapy. Overall, the results presented here expound a novel molecular mechanism for miR‐708 and CD47 in BCSCs and reveal that metformin plays an important role in this process. Moreover, the results might contribute to a better understanding of the present biological connection between breast cancer and type II diabetes.

## 2 MATERIALS AND METHODS

### 2.1 Cell lines and mammosphere culture

Human breast cancer cell lines MDA‐MB‐231 and MCF‐7 were obtained from American Type Culture Collection and cultured as previously described.^22^ All adherent cells were maintained at 37°C in a 5% CO_2_ incubator. For mammosphere culture in vitro, single cells were seeded into a 0.8% agarose‐coated 6‐well plate at a density of 3,000 cells per well with 2 mL MammoCult^TM ^medium containing 4 μg/mL heparin and 0.48 μg/mL hydrocortisone. Cells were incubated in a 5% CO_2_ incubator at 37°C for 10‐14 days, and mammospheres were counted under microscope with size >100 μm. For tumour inoculation in vivo, mammosphere cells that were obtained as described above were inoculated into the mammary fat pad of 4‐6 weeks old female BALB/c nude mice (obtained from Beijing Vital River Laboratory Animal Technology Company Limited) with or without miR‐708 overexpression (CD47 knockdown), and mice were monitored for tumorigenesis in the following one months. Metformin was used at 0.1‐10 mM and docetaxel (Sanofi Aventis) was used at 0‐1000 μM. All animal experiments in our study were approved by the Animal Ethics Committee of Sun Yat‐sen University and conducted in the Animal Center of Sun Yat‐sen University.

### 2.2 BCSCs marker analysis by flow cytometry

We assessed the expression of CD44 and CD24 surface markers after transfection with miR‐708 (or CD47 shRNA). Briefly, cells were washed once in PBS and stained with anti‐CD44 (APC‐conjugated; BD Biosciences) or anti‐CD24 (PE‐conjugated; BD Biosciences) antibody in PBS containing 1% FBS and incubated 4°C for 25 minutes. The cells were washed again with cold PBS and >10 000 cells were used for flow cytometric analysis (FACScalibur, BD Biosciences).

### 2.3 shRNA, lentiviruses and transduction

All lentiviral vectors in our study have a puromycin resistance gene. To overexpress or knockdown miR‐708 in cancer cells, about 500 bp of pri‐miRNA containing the mature miR‐708 sequence 5′‐AAGGAGCUUACAAUCUAGCUGGG‐3′ or the miRNA sponge (anti‐miR‐708) with sequence 5′‐CCCAGCTAGATCATAGCTCCTT‐3′ was amplified or synthesized, and then cloned into the lentiviral construct. Vectors encoding shRNA targeting CD47 were constructed based on the specific nucleotide sequences: shCD47: 5′‐CCG GGC TTC CAA TCAGAA GAC TAT ACT CGA GTA TAG TCT TCT GAT TGG AAG CTT TTT‐3′. Lentiviruses were packaged in 293T cells according to the manufacturer's instructions. Supernatant containing viral particles was collected 48 hours posttransfection, filtered (0.45‐μm pore size) and transduced into MDA‐MB‐231 and MCF‐7 cells in the presence of 8 μg/mL of Polybrene (Sigma‐Aldrich). After 24 hours, cells were maintained in medium containing 0.5 μg/mL puromycin (Sigma‐Aldrich).

### 2.4 MiRNA extraction and quantitative real‐time PCR

Total RNA and miRNA were isolated from cells and fresh tumour tissues using miReasy micro kit (Qiagen) and from paraffin‐embedded normal and malignant breast tissues using RecoverAll Total Nucleic Acid Isolation Kit (Ambion). A total of 500 ng of RNA were reverse transcribed in accordance with manufacturer's instruction (Superscript II reverse transcriptase, Invitrogen). miRNAs expression levels were quantified using TaqMan probes. Expression levels were normalized to those of RNU6B, and relative expression was calculated using the 2^ΔΔCt^ method.

### 2.5 Luciferase reporter assays

Wild‐type 3′ UTR of CD47 were amplified from genomic DNA by PCR. The mutant CD47 3′ UTR was synthesized using QuikChange Lightning Multi Site‐Directed Mutagenesis Kit (Agilent Technologies, Palo Alto, CA, USA). Both plasmids were then cloned into the pGL3‐control vector (Promega, Madison, WI, USA), and purified and finally co‐transfected with either scrambled miRNA or miR‐708 mimics into HEK293T cells using Lipofectamine 2000 (Invitrogen). The dual luciferase reporter assay was performed 24 hours after transfection using dual luciferase assay system (Promega) according to the manufacturer's protocol. All experiments were performed in triplicate.

### 2.6 Immunoblot assay

Cell lysates were prepared and the corresponding proteins were separated by SDS/PAGE, and probed with primary antibodies against CD47 (Novus Biologicals) and then HRP‐conjugated secondary antibodies (GE Healthcare) were used, with the anti‐actin antibody as an internel control (Santa Cruz).

### 2.7 Phagocytosis assay

Breast cancer cells were labelled with 5‐μM carboxyfluorescein succinimidyl ester (CFSE) and incubated for 15 minutes at 37°C in a CO_2_ incubator protected from light according to the manufacturer's protocol (Invitrogen). Macrophages were incubated in serum‐free medium for 2 hours before adding 2 × 10^5^ CFSE‐labelled breast cancer cells. After coculture at 37°C for 2 hours, cells were harvested, macrophages were stained with APC‐labelled anti‐F4/80 (Novus Biologicals), and phagocytosis were measured and analysed. Phagocytosis was calculated as the percentage of F4/80 + CFSE+ cells among CSFE + cells.

### 2.8 Patients and tissue samples

We enrolled 473 patients who underwent primary breast surgery for stage I–III invasive breast carcinoma in the Sun Yat‐sen University Cancer Center (SYSUCC). The study was approved by the Institutional Review Board of the SYSUCC and informed consent was obtained from all patients. For survival analyses, relapse free survivals in breast cancer patients were stratified by expression of the miR‐708 or CD47 expression and presented as Kaplan‐Meier plots. All clinical information of patients used in our study is summarized in Table 1.

**Table 1:** Clinicopathological variables and miR‐708 expression in 473 breast cancer patients

Characteristics	miR‐708 low expression (n = 281)		miR‐708 high expression (n = 192)		*P* value
	No.	%	No.	%	
Age (years)					0.223
<50	145	51.6	110	57.3	
≥50	136	48.4	82	42.7	
Tumour size (cm)					0.638
≤2	69	24.6	51	26.6	
>2	211	75.4	141	73.4	
Axillary lymph node					0.827
Non‐metastasis	125	45.3	85	44.3	
Metastasis	151	54.7	107	55.7	
Grade					0.079
I‐II	165	69.0	133	76.9	
III	74	31.0	40	23.1	
TNM stage					0.092
I‐II	164	59.0	128	66.7	
III‐IV	114	41.0	64	33.3	
ER status					0.305
Negative	188	68.6	123	64.1	
Positive	86	31.4	69	35.9	
PR status					0.326
Negative	193	70.4	127	66.1	
Positive	81	29.6	65	33.9	
HER2 status					0.380
Negative	236	86.8	170	89.5	
Positive	36	13.2	20	10.5	
CD47					**0.001**
Negative	125	44.5	116	60.4	
Positive	156	55.5	76	39.6	

% means percentage within the column.

Abbreviations: CD47, cluster of differentiation 47; ER, oestrogen receptor; HER2, human epidermal growth factor receptor 2; PR, progesterone receptor.

### 2.9 Statistical analysis

Data are expressed as the mean ± SD. Unless otherwise stated, Analyses of statistical significance were performed using the nonparametric Mann‐Whitney *U* test using the GraphPad Prism statistical program. The *P* values < 0.05 were considered. Error bars depict SD.

## 3 RESULTS

### 3.1 The expression of miR‐708 is markedly down‐regulated in BCSCs

**Figure 1:**
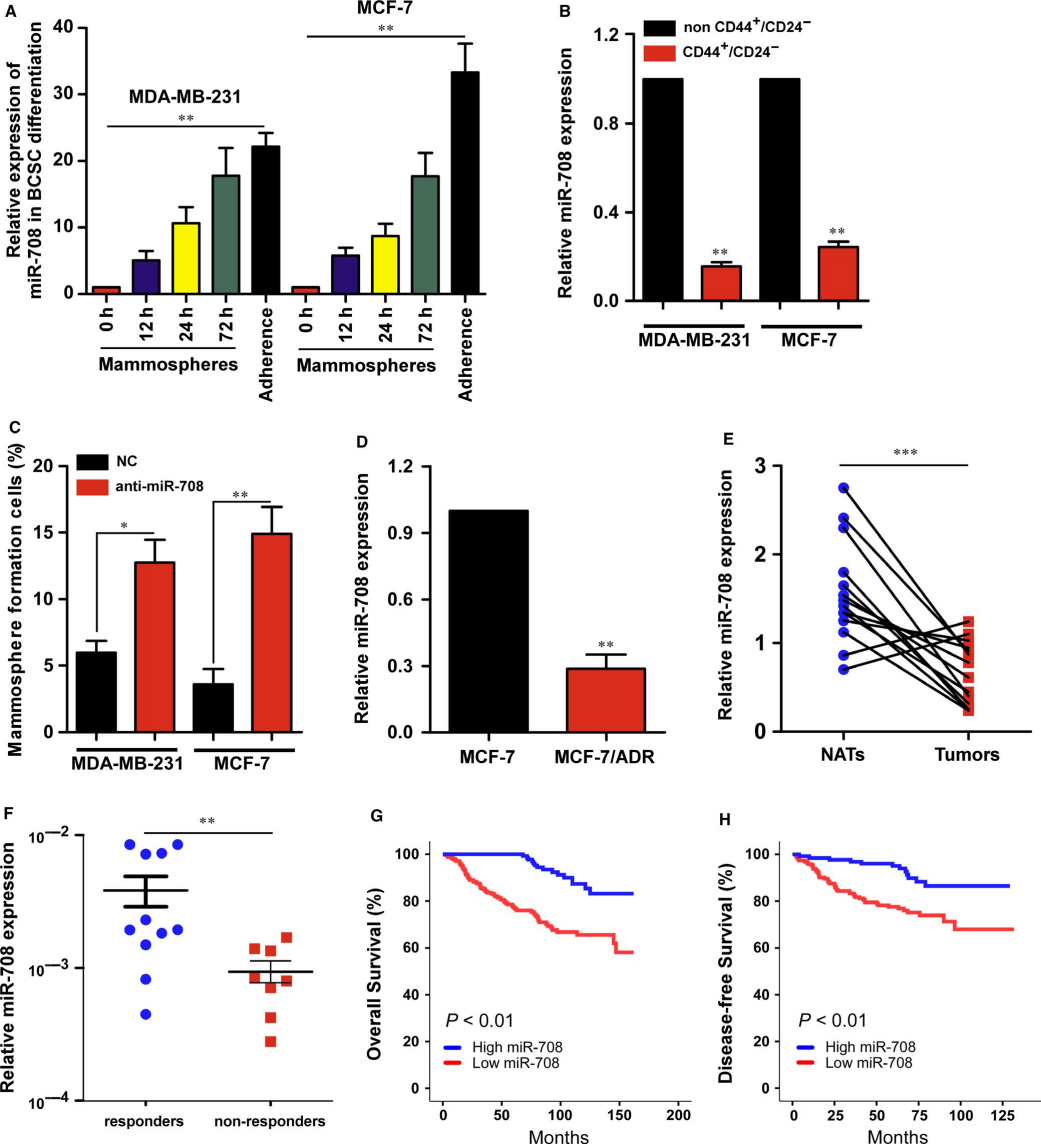
The expression of miR‐708 is markedly down‐regulated in BCSCs and correlated with patients’ prognosis. A, Expression of miR‐708 in spheres and adherent cells derived from MDA‐MB‐231 and MCF‐7 cells. B, Expression of miR‐708 in non CD44^+^/CD24^−^ and CD44^+^/CD24^−^ cells derived from MDA‐MB‐231 and MCF‐7 cells. C, Spheres formation assay of MDA‐MB‐231 and MCF‐7 cells treated with miR‐708 knockdown (anti‐miR‐708) or not (NC). Spheres were counted and compared on day 14. D, Relative expression of miR‐708 in MCF‐7 and MCF‐7/ADR cells. E, MiR‐708 expression was detected in breast cancer tumour tissues (Tumors) paired with normal adjacent tissues (NATs). F, MiR‐708 expression was detected in responders (n = 11) and nonresponders (n = 8) among patients with breast cancer receiving neoadjuvant chemotherapy. G and H, Kaplan‐Meier curves for OS (G) and DFS (H) of breast cancer patients with low vs high expression of miR‐708 in SYSUCC. 473 patients with breast cancer were divided into low miR‐708 (n = 281) and high miR‐708 groups (n = 192). NC, negative control; ADR, Adriamycin resistant; OS, overall survival; DFS, disease‐free survival; SYSUCC, sun yat‐sen university cancer center. *P < 0.05, **P < 0.01, ***P < 0.001

In many previous studies, CSCs have been shown to regulate the expression of some miRNAs.^23^, ^24^ In particular, the miR‐708 were reportedly down‐regulated in prostate CSCs.^11^ To evaluate whether BCSCs derived from MDA‐MB‐231 and MCF‐7 cells had a stronger self‐renewal capacity, we performed a sphere formation assay in vitro and a tumour initiation assay in vivo. First, we conducted qRT‐PCR analysis of mammospheres, mammospheres re‐adhered for 12, 24, 72 hours and adherent cells. As shown in Figure 1A, in MDA‐MB‐231 and MCF‐7 cell mammospheres differentiation, miR‐708 expression decreased significantly in mammospheres compared with re‐adhered mammospheres or adherent cells, which are associated with human cancer chemoresistance and metastasis. We then further examined the expression miR‐708 in non CD44^+^/CD24^−^ population (a marker of BCSCs) and CD44^+^/CD24^−^ population of MDA‐MB‐231 and MCF‐7 cells, and found miR‐708 was highly decreased in CD44^+^/CD24^−^ population compared with non CD44^+^/CD24^−^ population. In contrast, when miR‐708 was knocked down in adherent cell lines (MDA‐MB‐231 and MCF‐7), the mammosphere formation ability would be enhanced drastically. It was reported that chemoresistant cell lines had a better ability to form mammospheres and produce stem cell‐like property, and stemness‐related genes were often highly dysregulated. We then detected the miR‐708 expression in chemoresistant cell lines MCF‐7/ADR, and demonstrated that the expression of miR‐708 was significantly reduced in MCF/ADR cells compared with MCF‐7 cells. These results suggested that miR‐708 was significantly down‐regulated in BCSCs and affected sphere formation and chemoresistance (Figure 1A‐D).

### 3.2 MiR‐708 associates with breast cancer clinical characteristics and good prognosis

To investigate whether miR‐708 was involved in clinical breast cancer progression, we detected and analysed miR‐708 expression in breast cancer specimens. Remarkably, miR‐708 displayed down‐regulated in breast cancer tissues compared with normal tissues (Figure 1E). Moreover, miR‐708 was higher expression in neoadjuvant chemotherapy responders than non‐responders, indicating that miR‐708 might be associated with chemoresistance (Figure 1F). Furthermore, in patient samples, miR‐708 expression was not associated with conventional clinicopathological parameters (Table 1), however, Kaplan‐Meier survival analysis results showed that patients with high miR‐708 expression in breast cancers had significantly better overall survival (OS) and disease free survival (DFS) (Figure 1G,H). These data demonstrate that miR‐708 associates with chemosensitivity of breast cancer and may serve as a marker of good prognosis in breast cancer.

### 3.3 MiR‐708 inhibits the BCSCs

**Figure 2:**
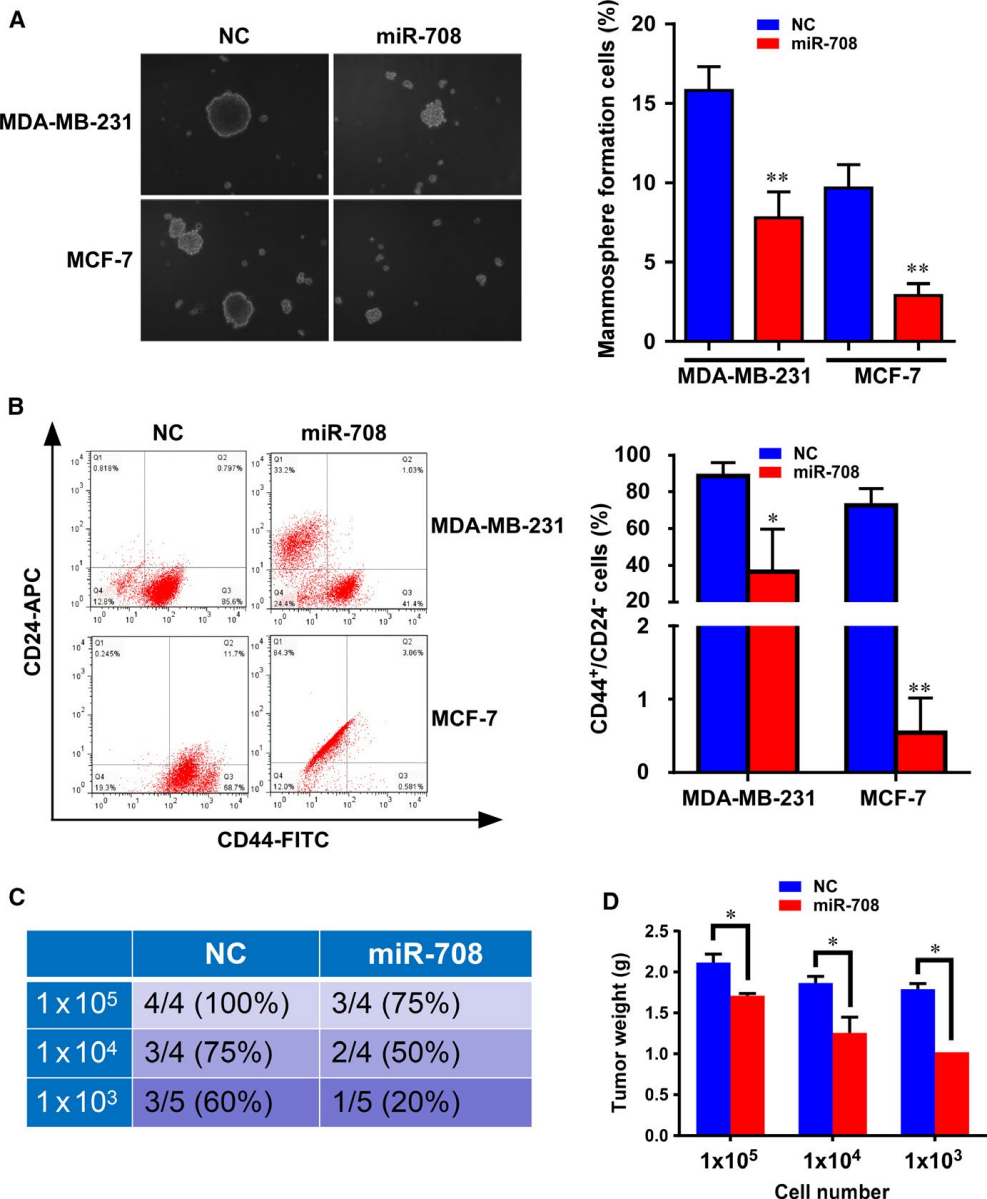
MiR‐708 effectively reduces the CD44^+^/CD24^−^ population and mammosphere formation of breast cancer cells. A. MiR‐708‐transfected MDA‐MB‐231 and MCF‐7 cells formed smaller mammospheres than cells transfected with NC, and the number of formed mammospheres were counted in 5 random fields on day 14. For mammosphere formation assay, 3000 cells were used, and mammospheres were counted with size >100 mm under microscope. B, Flow cytometry analyses of the CD44^+^/CD24^−^ population in MDA‐MB‐231 and MCF‐7 cells in mammosphere culture conditions using fluorescentconjugated CD44 and CD24 antibodies. The data represent the mean of 3 independent experiments. C, The numbers of animals with detectable tumours in the groups injected with the MDA‐MB‐231 mammosphere cells (with or without miR‐708 transfection before), were inoculated into the mammary fat pad of BALB/C nude mice. D, The detectable tumours in mice were measured for tumour weight one month later. NC, negative control. *P < 0.05, **P < 0.01

To assess the role of miR‐708 in cancer stemness, we evaluate the effect of miR‐708 overexpression on the self‐renewal capacity of BCSCs using a mammosphere formation assay. As expected, cells with miR‐708 overexpression formed fewer mammospheres than cells transfected with negative control (NC) in both MCF‐7.SC and MDA‐MB‐231.SC (Figure 2A). Mammospheres generated from NC groups were larger and grew more rapidly than those with miR‐708 overexpression groups. (Figure 2A). In addition, we examined the CD44^+^/CD24^−^ population following transfection with miR‐708 or NC. FACS analysis indicated that the overexpression of miR‐708 significantly reduced the CD44^+^/CD24^−^ population (Figure 2B). These results provide evidence that miR‐708 can inhibit the self‐renewal capacity of BCSCs with high efficiency. Furthermore, in order to address the effect of miR‐708 overexpression in BCSCs on tumour formation, we used the limited dilution and tumorigenesis assay in vivo. As shown in Figure 2C, for both the NC group and the miR‐708 overexpression group, 1 × 10^5 ^MDA‐MB‐231.SC cells formed tumour xenografts in nude mice with 100% efficiency, but the efficiency in the miR‐708 overexpression group is only 75%. When we decreased the cells to 1000 cells, tumours still formed in the NC group with 60% efficiency, but in overexpression group the efficiency decreased to 20% (Figure 2C). Additionally, miR‐708 overexpression significantly decreased the tumour weights (Figure 2D).

### 3.4 Identification of CD47 as a direct target of miR‐708

**Figure 3:**
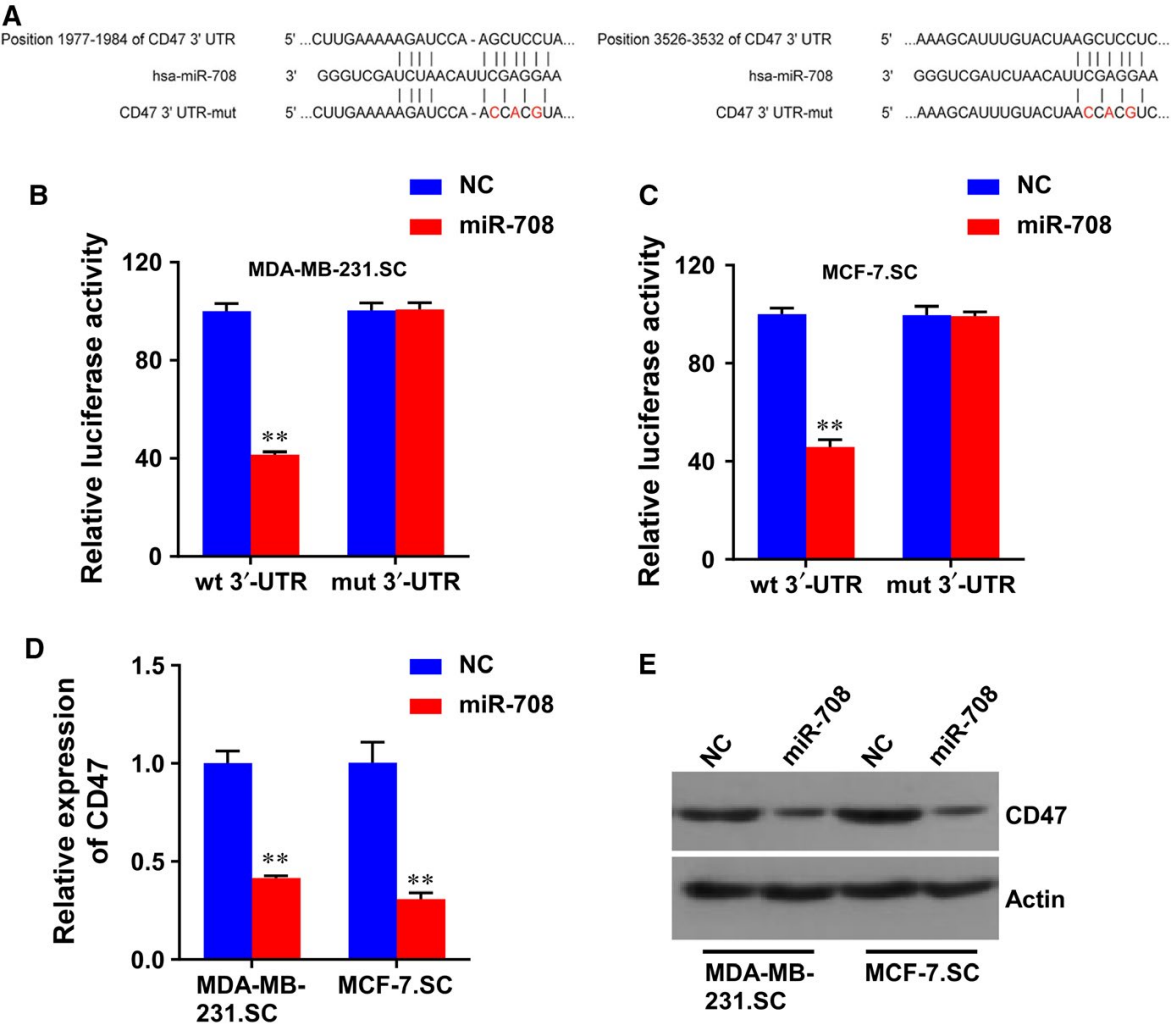
CD47 was a direct target of miR‐708 in breast cancer. A, Schematic of putative miR‐708 binding sequence in the 3′‐UTR of CD47, and the generation of mutation of CD47. B and C, Relative luciferase activity of CD47 wt or mut 3′‐UTR was detected in MDA‐MB‐231.SC and MCF‐7.SC transfected with miR‐708. D and E, CD47 expression was detected in the miR‐708 interfered MDA‐MB‐231.SC and MCF‐7.SC by real‐time PCR (D) and western blot (E). NC, negative control. **P < 0.01

To identify the target of miR‐708 that mediates the generation of stem cells in breast cancer, we searched for predicted target genes using miRanda (Figure 3A) and found the mirSVR score of CD47 ranked near the top in the obtained list. To further evaluate whether miR‐708 affects CD47 expression, we transfected MDA‐MB‐231.SC and MCF‐7.SC with miR‐708 and NC. The targeted role of miR‐708 on the 3′‐UTR of CD47 mRNA was assessed using luciferase assay. The luciferase activity were decreased in wide type CD47 3′‐UTR groups following miR‐708 overexpression, while that were not observed in the mutant type groups (Figure 3B,C). Moreover, we transfected miR‐708 into the stem cells and detected the CD47 expression. The mRNA levels of CD47 in the above cell lines were significantly decreased after ectopic miR‐708 expression (Figure 3D). In addition, an immunoblot analysis was also conducted, and data revealed that CD47 was expressed at a lower level in miR‐708 overexpression stem cells (MDA‐MB‐231.SC miR‐708 and MCF‐7.SC) than NC stem cells (Figure 3E). These results show that miR‐708 inhibits both mRNA and protein expression of CD47 in BCSCs.

### 3.5 shCD47 eradicates the BCSCs

**Figure 4:**
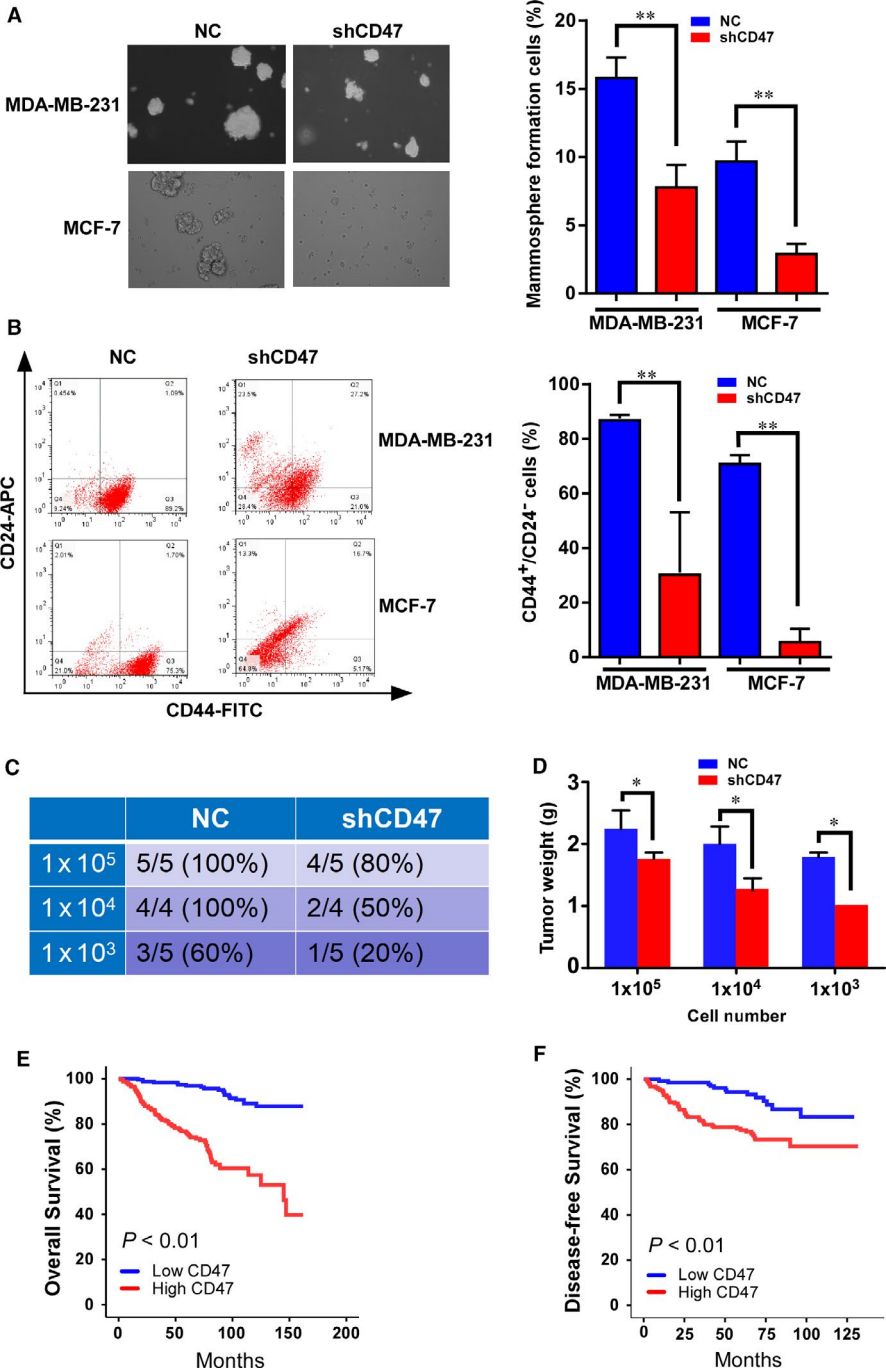
Knockdown of CD47 expression eradicates the BCSCs. A, MDA‐MB‐231 and MCF‐7 cells transfected with shCD47 formed smaller mammospheres than cells transfected with NC, and the number of formed mammospheres were counted in 5 random fields on day 14. For mammosphere formation assay, 3000 cells were used, and mammospheres were counted with size >100 mm under microscope. B, Flow cytometry analyses of the CD44^+^/CD24^−^ population in MDA‐MB‐231 and MCF‐7 cells transfected with shCD47 in mammosphere culture conditions using fluorescentconjugated CD44 and CD24 antibodies. The data represent the mean of 3 independent experiments. C, The numbers of animals with detectable tumours in the groups injected with the MDA‐MB‐231 mammosphere cells (with or without shCD47 transfection before), were inoculated into the mammary fat pad of BALB/C nude mice. D, The detectable tumours in mice were measured for tumour weight 1 month later. E and F, Kaplan‐Meier curves for OS (E) and DFS (F) of breast cancer patients with low vs high expression of CD47 in SYSUCC. A total of 473 patients with breast cancer were divided into low CD47 (n = 241) and high CD47 groups (n = 232). NC, negative control. *P < 0.05, **P < 0.01

To further assess the role of CD47 in BCSCs, we constructed shRNA targeting CD47 (shCD47), and evaluate the effect of knockdown of CD47 expression on the self‐renewal capacity of BCSCs. As expected, cells with CD47 knockdown formed fewer mammospheres than cells transfected with NC in both MCF‐7.SC and MDA‐MB‐231.SC (Figure 4A). Mammospheres generated from NC groups were larger and grew more rapidly than those from shCD47 groups. (Figure 4A). In addition, we examined the CD44^+^/CD24^−^ population following transfection with shCD47 or NC. FACS analysis indicated that the knockdown of CD47 expression significantly reduced the CD44^+^/CD24^−^ population (Figure 4B). These results provide evidence that CD47 knockdown can suppress the self‐renewal capacity of BCSCs with high efficiency. Moreover, we used the limited dilution assay in vivo to evaluate the effect of CD47 in BCSCs on tumour formation. In the CD47 knockdown group, 1 × 10^5 ^MDA‐MB‐231.SC cells formed tumour xenografts in nude mice with 80% efficiency, while the efficiency in the NC group is 100%. When we decreased the cells to 1000 cells, tumours formation efficiency decreased in both groups, especially in shCD47 group with 20% (Figure 4C). Furthermore, knockdown of CD47 expression significantly decreased the tumour weights (Figure 4D). Thus, CD47 loss‐of‐function contributed to eradicate the BCSCs. Furthermore, considering that miR‐708 was demonstrated to be associated with breast cancer clinical characteristics and good prognosis, we then analysed clinical relevance of CD47 in breast cancer. Interestingly, The CD47 level in primary breast tumours was significantly positive correlation with miR‐708 expression (Table 1) and patients’ poor survival (Figure 4E,F).

### 3.6 CD47 deficiency and miR‐708 overexpression increase the phagocytosis of macrophages and chemosensitivity in breast cancer

**Figure 5:**
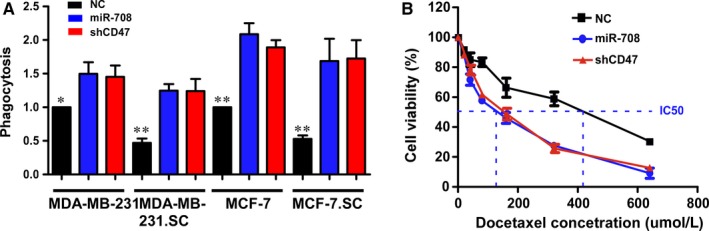
CD47 deficiency or miR‐708 overexpression can increase the phagocytosis and chemosensitivity of breast cancer cells. A, The MDA‐MB‐231, MDA‐MB‐231.SC, MCF‐7 and MCF‐7.SC cells were transfected to NC, shCD47 and miR‐708 for 48 h, incubated with bone marrow‐derived macrophages for 2 h, and the percentage of phagocytosed cancer cells was determined and normalized to the NC at adherent cells. B, Cell viability was measured through CCK‐8 assay to evaluate the chemosensitivity of shCD47 and anti‐miR‐708 in MDA‐MB‐231. NC, negative control. *P < 0.05, **P < 0.01

We observed decreased expression of CD47 in stem cells treated with miR‐708 (Figure 3D,E), leading us to hypothesize that miR‐708 overexpression may promote phagocytosis of BCSCs by macrophages. To test this hypothesis, we performed in vitro phagocytosis assays on breast cancer cells and stem cells. We firstly transfected MDA‐MB‐231, MDA‐MB‐231.SC, MCF‐7 and MCF.SC cells with miR‐708 and shRNAs targeting CD47 (shCD47), cultured with bone marrow‐derived macrophages for 2 hours, and found that phagocytosis was significantly increased in miR‐708 overexprssion and shCD47 groups compared with NC, in the above four cell lines (Figure 5A). CSC culture condition modestly decreased phagocytosis of both MDA‐MB‐231 and MCF‐7 cells, which suggests the phagocytic function of macrophages impaired by CSCs can be reversed by miR‐708‐CD47 signal pathway (Figure 5A).

Next, we examined the chemosensitivity of the MDA‐MB‐231.SC NC, MDA‐MB‐231.SC miR‐708 and MDA‐MB‐231.SC shCD47 cells to docetaxel (Figure 5B). Downregulation of CD47 or overexpression of miR‐708 enhanced the sensitivity of MDA‐MB‐231 cells to docetaxel. The IC50 value of docetaxel towards the MDA‐MB‐231.SC miR‐708 cells was approximately threefold lower than that towards the MDA‐MB‐231.SC NC cells (Figure 5B). Taken together, these results demonstrate that downregulation of CD47 or overexpression of miR‐708 improved sensitivity of MDA‐MB‐231 cells to docetaxel.

### 3.7 MiR‐708/CD47 signal pathway is a target of metformin

**Figure 6:**
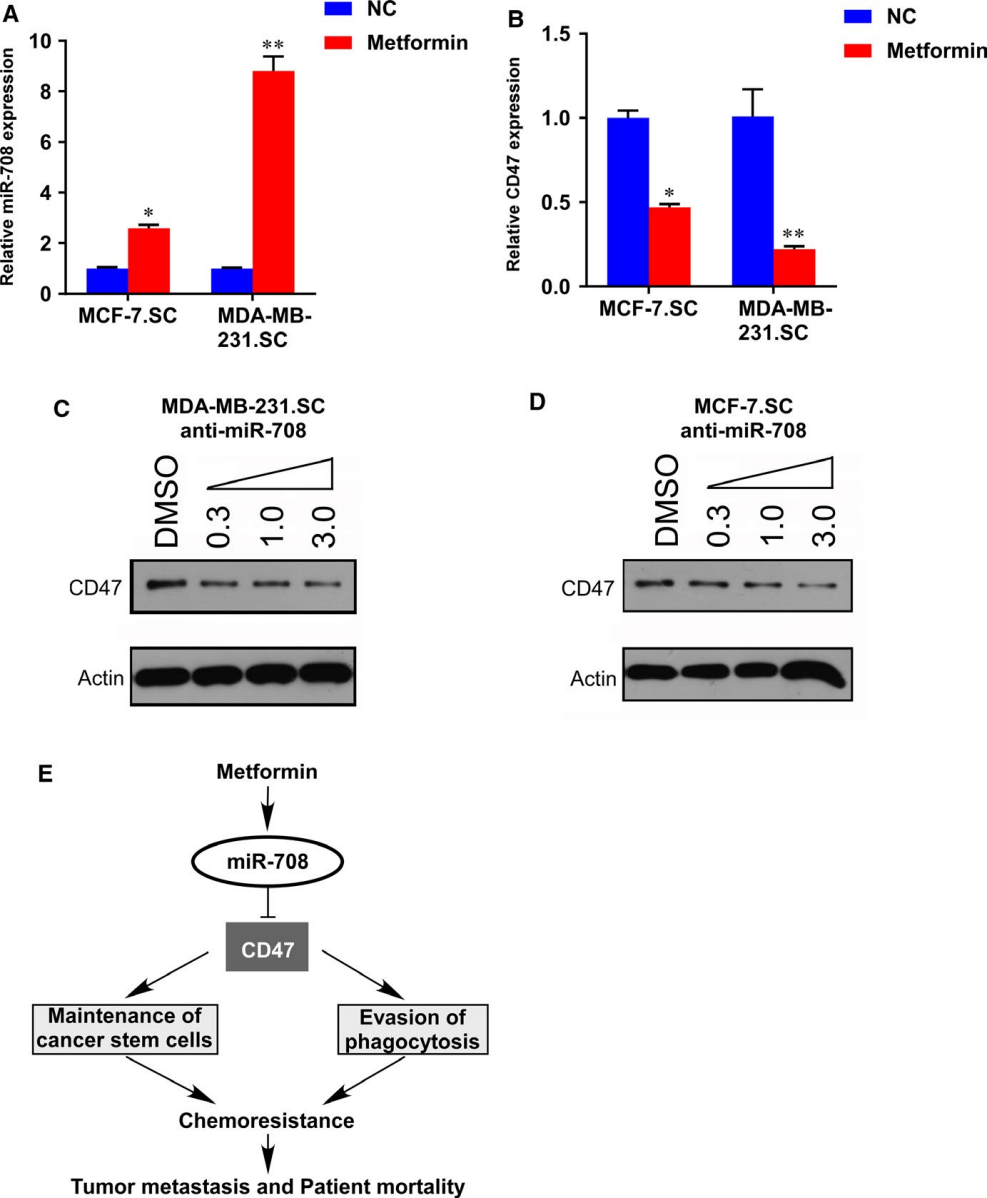
Metformin induces miR‐708‐mediated suppression of CD47. A and B, Metformin increased the miR‐708 (A) and decreased the CD47 (B) expression level in MDA‐MB‐231.SC and MCF‐7.SC. The cells were treated with 10 mM metformin or PBS for 48 h and the mRNA levels were determined by quantitative RT‐PCR. The statistical significance was determined by student's *t* test. C and D, Immunoblot analyses of CD47 expression in MDA‐MB‐231.SC anti‐miR‐708 (C) and MCF‐7.SC anti‐miR‐708 (D) cells incubated with metformin (0.3‐3.0 mM) for 72 h. β‐actin was used as a loading control. E, A schematic model of the mechanism underlying the role of metformin and miR‐708 in BCSCs self‐renewal and chemoresistance. *P < 0.05, **P < 0.01

Previous studies showed that metformin can selectively targets BCSCs and suppress breast tumour growth.^21^, ^25^, ^26^ In addition, CD47 has been reported to show different expression levels in the development of type II diabetes mellitus.^27^ Then we hypothesized that metformin may attenuate the BCSCs through miR‐708‐mediated suppression of CD47. To test this hypothesis, we firstly evaluated the miR‐708 and CD47 expression in MCF‐7.SC and MDA‐MB‐231.SC cells treated with metformin for 48 hours, and found that miR‐708 expression were all significantly elevated, while CD47 mRNA expression were down‐regulated in both cell lines (Figure 6A,B). Moreover, we subsequently examined the protein expression levels of CD47 in MDA‐MB‐231.SC anti‐miR‐708 and MCF‐7.SC anti‐miR‐708 cells that were incubated with 0.3 mM, 1.0 mM and 3.0 mM metformin for 72 hours. The protein expression levels revealed a dose‐dependent suppression of CD47 expression by metformin in both cell lines (Figure 6C,D). These results suggest that CD47 is a novel target of metformin in breast cancer cells and that metformin attenuates stem cells through miR‐708‐mediated suppression of CD47.

## 4 DISCUSSION

The self‐renewal and differentiation of BCSCs are affected by multiple transcriptional regulatory circuits. Although many studies have focused on the molecular mechanism of CSC, there are still many pending issues so far.^5^, ^21^, ^23^, ^28^ To clarify the intrinsic regulatory network of BCSCs is a prerequisite for developing more effective cancer treatment. In this study, we have demonstrated that miR‐708 regulates the production of stem cells in breast cancer that shows docetaxel resistance and high tumorigenicity. The CD47 gene that is a negative modulator of insulin receptor activation and associated with type II diabetes mellitus development,^27^ was identified as a direct target of miR‐708. CD47 induced the production of stem cells and phagocytosis of macrophages, leading to chemoresistance of breast cancer. In addition, mammosphere culture conditions that enrich BCSCs induced downregulation of miR‐708 and overexpression of CD47. Furthermore, metformin, a first line drug for type 2 diabetes mellitus, decreased the generation of stem cells through miR‐708‐mediated suppression of CD47.

CSCs are the main cause of drug resistance of tumours, which can lead to cancer recurrence or metastasis, thus affecting the poor prognosis of patients.^19^, ^21^, ^28^ Therefore, if CSCs population are the main driving force of cancer initiation and mediate drug resistance in general chemotherapy, then approaches that target this CSCs could improve the effectiveness of treatment regimens and abate the risk of cancer recurrence. Several miRNAs have already been deemed as ‘‘fine‐tuners’’ of stem cell fate and possess anti‐cancer effects.^23^ Here, down‐regulation of miR‐708 was observed in breast cancer patients and up‐regulation of miR‐708 expression induced a selective reduction in the stem cells that showed drug resistance and high tumorigenic activity. Therefore, given the close relationship between the clinical behaviour and the CSCs prosperity of breast cancer, our result opens an avenue to develop new therapeutic strategies targeting the miR‐708/CD47 axis.

In recent years, immune function for cancer treatment, including breast cancer, have been increasingly recognized and many attempts have been made, mainly by enhancing immune response or suppressing immune evasion.^12^, ^13^, ^14^ Therefore, it is important to elucidate the relevant mechanisms, because immunotherapy may improve the management of cancer patients. Tumour cells avoid immune surveillance by interacting directly or indirectly with various immune cells. Recently, much attention has been focused on modifying immune cell responses as a basis for developing new cancer treatments.^12^, ^19^ CD47 is a cell surface protein that interacts with signal‐regulated protein alpha on macrophages to block phagocytosis.^14^, ^15^, ^16^ It is the main mechanism that mediates cancer cells to escape innate immunity. Prior studies have demonstrated that the expression of CD47 in hypoxic breast cancer cells can be induced by HIF‐1 and chemotherapy.^19^, ^20^ However, whether CSCs also affects the expression of CD47 has not been reported in breast cancer. In our study, we observed that mammosphere cells derived from adherent cells can stimulate the CD47 expression. In addition, CD47 was also identified as a novel target of miR‐708 that controls the phagocytosis activity of macrophages by regulating its expression. The results presented here also demonstrate that CD47 regulates the drug resistance of the stem cells in breast cancer. What is more, the expression of CD47 was elevated in breast cancer patients with poor prognosis, then incorporating CD47 into our clinicopathological staging system may be useful in future.

Type 2 diabetes mellitus has been reported to be associated with increased incidence and mortality of a number of malignancies, including breast cancer.^29^, ^30^, ^31^, ^32^, ^33^ Previous study results have displayed that metformin selectively blockades the inflammatory pathway in BCSCs by inhibiting signal transducers and activators of nuclear factor‐kB, thus inhibiting BCSCs phenotypes.^21^, ^34^ However, the molecular mechanism of metformin restoring chemosensitivity of CSCs remains unclear. We stated the fact that metformin regulates miR‐708/CD47 axis to eradicate BCSCs and enhance chemosensitivity. It should be noted that we could not elucidate the molecular mechanism and the role of CD47 in the acquisition of tumour seeding ability; therefore, additional investigations of CD47 functions are required. However, the results of our study may provide a new molecular mechanism for the anti‐BCSCs effect of metformin.

To sum up, the function of miR‐708 in increasing chemosensitivity and attenuating tumour stem cells suggests that RNA‐based treatment may improve the outcome of breast cancer patients while routine chemotherapy is performed. Moreover, our study has some implications for raising the existing understanding of biological relationships between breast cancer initiation and type 2 diabetes mellitus development.

## ETHICS APPROVAL AND CONSENT TO PARTICIPATE

This study was approved the Institutional Review Board of Sun Yat‐sen university cancer center (GZR2013‐108).

## CONSENT FOR PUBLICATION

We have received consents from individual patients who have participated in this study. The consent forms will be provided upon request.

## CONFLICT OF INTERESTS

We declare that we have no conflict of interest.

## Data Availability

The datasets supporting the conclusions of this article are included within the article and its Additional files.
